# A Novel Dielectric Barrier Discharge (DBD) Reactor with Streamer and Glow Corona Discharge for Improved Ozone Generation at Atmospheric Pressure

**DOI:** 10.3390/mi12111287

**Published:** 2021-10-21

**Authors:** Pu Liu, Yongxin Song, Zhitao Zhang

**Affiliations:** Department of Marine Engineering, Dalian Maritime University, Dalian 116026, China; yongxin@dlmu.edu.cn (Y.S.); new_zhangzhitao@163.com (Z.Z.)

**Keywords:** dielectric barrier discharge, silver layer, ozone synthesis, glow corona discharge

## Abstract

Discharge mode is an important parameter for ozone synthesis by dielectric barrier discharge (DBD). Currently, it is still challenging to stably generate glow discharge with oxygen at atmospheric pressure. In this paper, a DBD reactor with a layer of silver placed between the electrode and the dielectric layer (SL-DBD) was developed. Experimental results show that both streamer and glow corona discharge were stably generated under sinusoidal excitation with a 0.5 mm discharge gap in a parallel-plate DBD, due to the increased electric field strength in the discharge gap by the silver layer. It was also found that, in the SL-DBD reactor, glow corona discharge enhances the discharge strength by 50 times. The spectral peak of O at 777 nm in SL-DBD is increased to 28,800, compared with 18,389 in a reactor with a streamer only. The SL-DBD reactor produces ozone with a concentration of as high as 150 g/m^3^ and shows good stability in an 8 h durability test.

## 1. Introduction

Dielectric barrier discharge (DBD) plasma is an efficient method for ozone synthesis, which has been widely used for air pollution control [1,2,3,4,5] and water treatment [6,7,8,9,10]. For DBD, discharge intensity within the discharge gap is an important parameter for ozone synthesis. Depending on the electric field intensity in the discharge gap, different discharge modes, such as Townsend discharge, streamer discharge, corona discharge, and glow discharge can be generated. Townsend discharge refers to electrons collide with gas molecules and produce new electrons. The intensity of Townsend discharge is very low, and it is difficult to convert oxygen into ozone. Streamer discharge refers to cations that develop and continuously ionize from the anode to the cathode. The intensity of streamer discharge is very high, and it can convert oxygen into zones, which is also recognized as the working principle of the DBD reactor. Corona discharge and glow discharge are usually used for negative discharge [11,12,13,14,15,16]. Their discharge intensity is lower than that of a streamer. It is generally agreed that a stable discharge with high intensity is favorable for ozone synthesis.

To increase discharge intensity, Elkholy et al. [17] conducted time-resolved electrical and optical measurements to characterize the main features of the plasma discharge in the DBD micro-plasma reactor. The micro-plasma reactor consists of 363 parallel channels with a diameter of 400 μm. Under atmospheric pressure and 50 mbar, the pulse energy of each channel is 1.46 μJ and 1.3 μJ. It was found that the discharge at low pressure is characterized by a high vibrational temperature (roughly 4000 K) and high electric field strength (1000 Td), compared with atmospheric pressure (3460 K and 550 Td, respectively), which indicates higher electron energy at lower pressure. Li et al. [18] designed a highly efficient discharge reactor, which has a fence-like electrode in one thin dielectric layer and allows reactant gas to flow through the two plasma zones in sequence while the electron excitation temperature reaches 0.7 eV. This experimental result shows that the use of grid electrodes can produce high-intensity discharge near the electrode, and the electron temperature can reach 0.7 eV. Lu et al. [19] developed a DBD reactor with TiO_2_ thin film to improve the discharge intensity, as well as the number of reactive species and charges accordingly. It can be seen that adding a catalyst to the surface of the dielectric layer is an effective method to increase the discharge intensity. Zhao et al. [20] reported a packed-bed DBD reactor with glass beads for gaseous NOx removal. It was found that the intensity of discharge was enhanced. This is because the dielectric beads alter the distribution of the electric field due to the polarization at the glass bead surfaces. It should be noted that the method of changing the gas pressure, electrode shape, and adding catalyst or dielectric beads can effectively increase the electric field strength. However, whether the discharge modes changes in the reactor has not been studied.

As is well known, the electric field strength of the discharge gap changes the discharge mode. Abdelaziz et al. [21] investigated the effect of discharge electrode spike on discharge mode. The results showed that oxygen DBD is effective in the streamer mode at all frequencies and at atmospheric pressure. Li et al. [22] found that the discharge mode changes from Townsend discharge to glow discharge as the electric field strength increases under sinusoidal excitation. It was also found that under sinusoidal excitation at atmospheric pressure, the discharge mode is changed to a glow corona discharge from the pattern discharge as the electric field strength changes [23]. Yu [24] found that at 3 kV in needle-plate DBD, streamer discharge is formed in the positive half-cycle. For the negative half-cycle, corona or Trichel pulse discharge is generated. The discharge gap is 0.9 mm, and the thickness of the dielectric layer is 0.47 mm. The material of the dielectric layer is Al_2_O_3_. When the voltage is increased to 6 kV, the positive half-cycle of discharge is a streamer, and the negative half-cycle of discharge is glow discharge. Moreover, three kinds of DBD devices were designed to compare the effects of different discharge modes. The results showed that streamer and glow discharge generate alternately only when the dielectric layer is covered on the ground electrode. For the double dielectric layer structure, there is only streamer discharge. However, the above investigations were carried out only in small-scale experimental systems, not in ozone reactors.

When the electric field strength in the discharge gap is increased, however, side effects such as partial discharge occurs at the contact surface between the dielectric layer and the electrode.

As reviewed above, it is still challenging to generate stable hybrid discharges with high-intensity in ozone reactors. In this paper, a DBD reactor with a layer of silver placed between the electrode and the dielectric layer (SL-DBD) was developed to increase the electric field strength in the discharge gap without partial discharge side effects. The effects of the electric field strength and discharge modes on ozone synthesis were systematically investigated. The stability testing of the reactor was also performed.

## 2. Materials and Methods

### 2.1. Experimental System

**Figure 1:**
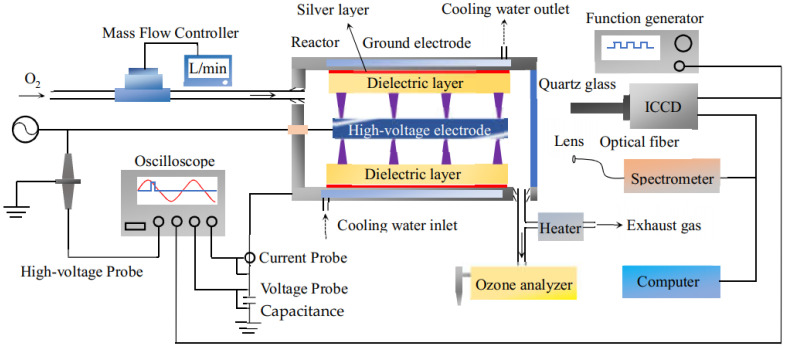
Schematic diagram of the experimental setup.

Figure 1 shows the components and working principles of the DBD experimental system. The system can be divided into three parts. The first is the gas circuit unit, which consisted of feed gas (oxygen, 99.6% purity), a mass flowmeter (Laifeng LF 400-S/A, Chengdu, China), and a heater. This unit provided reaction gas and treat the exhaust gas. The second part is the discharge unit including the SL-DBD reactor and cooling system. The reactor converted oxygen into ozone by generating high-intensity discharge. Cooling water (4.8 °C) was circulated in the reactor shell to decrease the working temperature of the reactor during the experiment. The ground electrode was designed as a hollow structure, in which cooling water was circled to reduce the gas temperature in the discharge gap and improve the ozone concentration simultaneously. The third part is the detection system, which was used to monitor the discharge parameters. The experiment mainly included the equipment shown in Figure 1.

The concentration of generated ozone in the reactor was measured in real time by an ozone analyzer (BMT-964, Berlin, Germany). The exhaust gas was heated with a heater (Suhai Heating Equipment SH-TRO, Yancheng, China) before being released into the air. At the same time, the power supply was self-made and consisted of an insulated-gate bipolar transistor (IGBT) inverter and transformer. The design of IGBT inverter is shown in the supplementary. The input voltage is 220 V/50 Hz, the output voltage range is 0–6000 V, the output frequency range is 4-10 kHz, and the waveform of the output is a sine. The current probe (Tektronix P6022, Beaverton, OR, USA) and the voltage probe (Tektronix P6015A, Beaverton, OR, USA) were connected to an oscilloscope (Tektronix DPO4104, Beaverton, USA) for detecting the current-voltage waveform. A 550nF capacitance was inserted in the circuit, and a voltage probe (Tektronix TPP0500B, Beaverton, OR, USA) was connected to the capacitance and oscilloscope so that the Lissajous figure was recorded.

In addition, the discharge image was taken by an intensified charge-coupled device (ICCD) camera (ANDOR DH334T, Belfast, UK) equipped with a macro lens (CCS SE16SM, Tokyo, Japan) through the quartz glass. The ICCD was triggered by a function generator (Tektronix AFG 3021, Beaverton, OR, USA). The light emission during the discharge was collected by the optical fiber with a lens and transmitted into the entrance slit of a spectrometer (ACTON SP2758, Trenton, NJ, USA). The width of the entrance slit of the spectrometer was 0.05 mm, and the integration time of the ICCD was set as 3 milliseconds.

### 2.2. Structure and Fabrication of the Reactors

**Figure 2:**
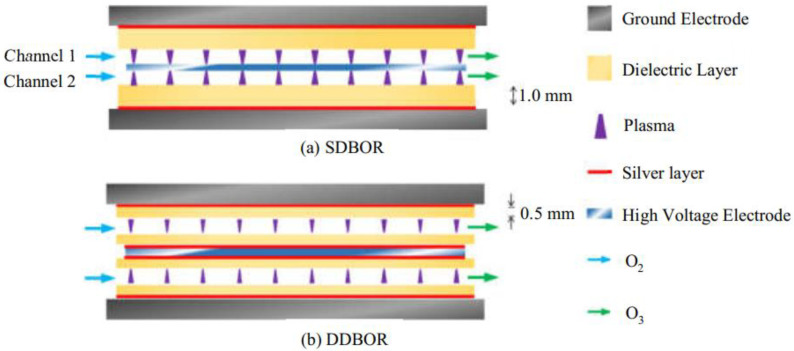
Internal structure of dielectric barrier discharge with silver layer (SL-DBD) reactor with double channel. (**a**) a single dielectric-barrier ozone reactor (SDBOR); (**b**) double dielectric-barrier ozone reactor (DDBOR).

In this study, two different SL-DBD reactors were investigated. One is named a single dielectric-barrier ozone reactor (SDBOR, Figure 2a) in which silver layers were placed only between the ground electrodes and the dielectric layer with a thickness of 1.0 mm. The other one is named a double dielectric-barrier ozone reactor (DDBOR, Figure 2b) in which silver layers were placed between the high voltage electrode and the dielectric layers, and between the ground electrode and the dielectric layers with a thickness of 0.5 mm. For each reactor, the total discharge volume within each channel is 11.2 cm^3^ (190 mm × 118 mm × 0.5 mm, length × width × height). The total discharge volume for each reactor is therefore 22.4 cm^3^.

**Figure 3:**
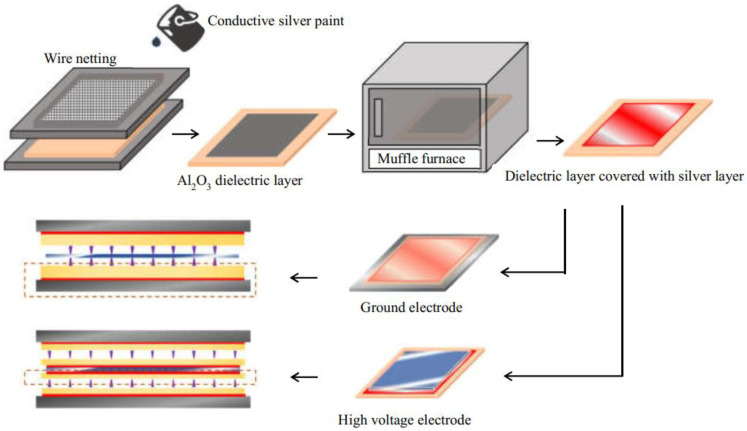
Fabrication of dielectric layer covered with silver layer.

To fabricate the silver layer between the electrode and dielectric layer, firstly, a wire mesh was put on the surface of the dielectric layer. Afterward, the conductive silver paint was carefully poured on the wire mesh to make the conductive silver paint uniformly covered on the surface of the dielectric layer. Then, the dielectric layer with the silver layer was slowly heated to 650 °C for 4 h in a muffle furnace and cooled naturally, as shown in Figure 3. This prevented the dielectric layer from being deformed due to sudden heating or cooling. Thus, the dielectric layer with a silver layer was fabricated. The thickness of silver layers is less than 0.03 mm.

Once a silver-coated dielectric layer was obtained, the next step is to place a Polytetrafluoroethylene (PTFE) cushion strip with a thickness of 0.5 mm and width of 10 mm between the dielectric layer and the ground electrode to ensure that the discharge gap was 0.5 mm. The final step was to seal the reactor with glue and fix it with 4 screws.

In the SDBOR, the dielectric layer covered with a silver layer was put on the ground electrode. For the DDBOR, on the contrary, two silver-covered dielectric layers were put on the high voltage electrode. The other silver-covered dielectric layer was put on the ground electrode. The high voltage electrode and ground electrode were designed as parallel plates in the SL-DBD reactor.

### 2.3. Experimental Procedures

To begin an experiment, first, oxygen (99.6% purity) was added into the reactor for 2 min to ensure that there was no other gas in the reactor. Afterward, the applied voltage was slowly increased to 2.8 kV. At the same time, current–voltage waveforms and optical emission spectroscopy were recorded, and discharge images were taken. The exposure time was set to 10 milliseconds, and the discharge image was superimposed 1000 times. Ozone concentration was measured at an interval of 30 min.

## 3. Results and Discussion

### 3.1. Discharge Modes

Theoretically, the electric field strength in the discharge gap will influence discharge modes, which, in turn, will influence ozone synthesis. Thus, it is of great interest to know the discharge modes in the two novel SL-DBD reactors (Figure 2).

**Figure 4:**
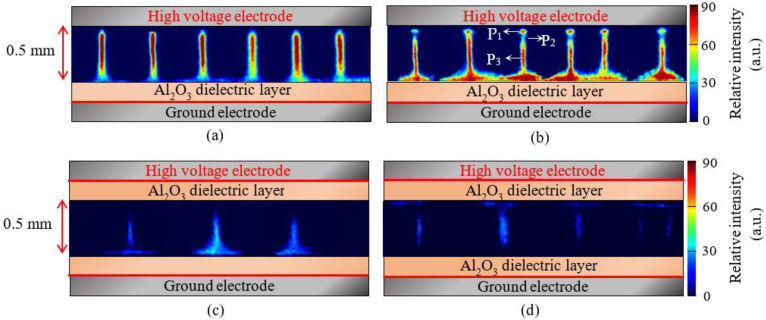
Typical discharge images in different cycles: (**a**) SDBOR in the positive half-cycle; (**b**) SDBOR in the negative half-cycle; (**c**) DDBOR in the positive half-cycle; (**d**) DDBOR in the negative half-cycle, (applied voltage = 2.8 kV, discharge gap = 0.5 mm, exposure time = 1 μs).

Figure 4 shows the typical discharge images of the two reactors. For the SDBOR, from Figure 4a,b, it can be found that the discharge modes in the two half-cycles are different. In the positive half-cycle (Figure 4a), there are several independent discharges in the discharge gap, and each discharge is characterized with vertical straight lines, which are arranged uniformly in the discharge gap. For each discharge line, it is continuous in the vertical direction and has no disconnected part. Obviously, the discharge mode is a typical streamer discharge. The reason for generating such a discharge mode is that electrons are absorbed by the anode, and the ions gathered near the high voltage electrode surface are pushed toward the dielectric layer. This will form an intrinsic electric field, which is in the same direction as the applied electric field. When the magnitude of the two electric fields (the applied and the intrinsic electric field) is greater than that of the breakdown electric field, a streamer discharge is generated.

With regard to the negative half-cycle, as is shown in Figure 4b, it is clear that the form of discharge is obviously different from streamer discharge. Streamer discharge is shown as a continuous state in the discharge image, while the negative half-cycle discharge is discontinuous and consists of a negative glow (P_1_), Faraday dark (P_2_), and positive column (P_3_). It can be inferred that this is an obvious glow corona discharge.

The glow corona discharge mode in the negative half-cycle is due to the strong electric field near the cathode. In the negative half-cycle, an electric field is formed by the deposited charges in the positive half-cycle and superimposed with the applied electric field. As a result, a stronger electric field is formed near the cathode, resulting in a higher cathode potential drop and a brighter negative glow, as shown in Figure 4; thus, (P_1_) is formed. After an inelastic collision, electrons lose a lot of energy and cannot be excited and ionized. Therefore, a dark area is formed, which is the Faraday dark (P_2_). When the electrons are accelerated again by the applied electric field, the columnar area (P_3_) is formed. The corresponding ICCD images of SDBOR is shown in Figure S4 of the supplementary.

For the DDBOR, in the positive half-cycle (Figure 4c), the discharge is formed at a certain distance away from the anode and spread in a form of a straight line. When the discharge reaches the surface of the dielectric layer, the discharge spreads to both sides. Obviously, this is streamer discharge. The reason is that the electrons in the discharge are continuously excited, and their intensity is continuously enhanced.

The reason why the discharge deflects to both sides is that the accumulation of charges on the surface of the dielectric layer will form electron clusters. The electric field formed by the electron clusters will deflect the discharge to both sides.

Figure 4d shows the discharge of the DDBOR in the negative half-cycle. It is clear that the discharge mode is the same as that in the positive half-cycle (Figure 4c), and different from that in the SDBOR negative half-cycle (Figure 4b). It should be noted that the direction of discharge is opposite to that in the positive half-cycle, which is due to the reversed positions of the cathode and anode. Compared with SDBOR, such a streamer discharge in the DDBOR is due to the electron clusters formed by electron avalanche is not absorbed by the unexposed high voltage electrode (anode).

In summary, SL-DBD reactors with different structures can produce different discharge modes. SDBOR reactor can generate streamer discharge and glow corona discharge. However, the DDBOR reactor can only generate streamer discharge modes. The influence of the silver layer on the discharge modes and the ozone generation is analyzed in the following sections.

### 3.2. Effect of Discharge Modes on Current-Voltage Waveforms and Lissajous Figure

**Figure 5:**
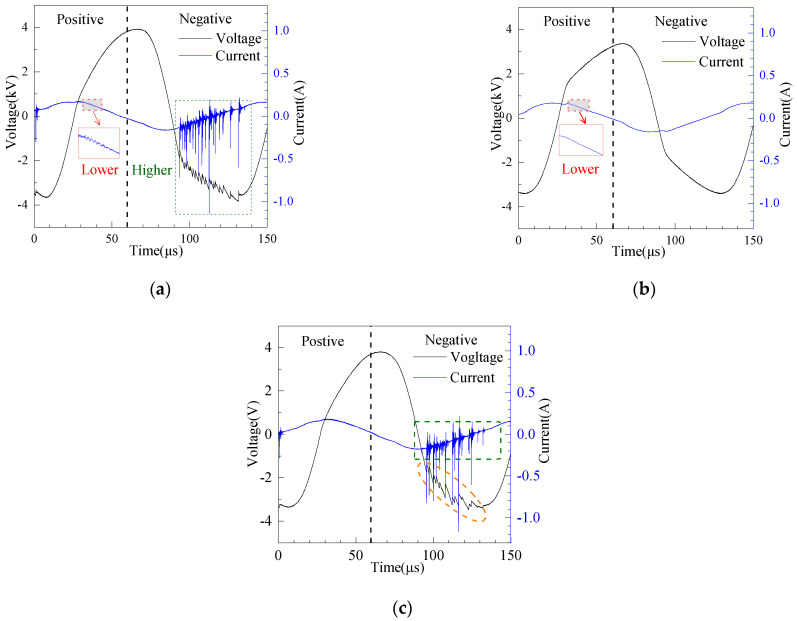
Current–-voltage waveforms of SDBOR (**a**), DDBOR (**b**), and DBD reactor without silver layer (**c**) (discharge gap = 0.5 mm, applied voltage = 2.8 kV, the marked positive and negative refers to current).

Generally, the discharge intensity and discharge power of a DBD reactor are evaluated by the current-voltage waveforms and Lissajous Figure. Figure 5a shows the current-voltage waveforms of the SDBOR. It can be found that there are many current pulses with small magnitude in the positive half-cycle, and many higher-magnitude current pulses in the negative half-cycle, as indicated by the red and green dashed boxes in Figure 5a. The current-voltage waveform of the DDBOR is shown in Figure 5b. It is clear that the positive and negative half-cycles of the current waveforms consist of many current pulses with low magnitude. The reason for the above difference is that the discharge modes in the two cycles are different. In the parallel-plate DBD, the displacement current strength is enhanced, thus covering most of the streamer current pulse. It can also be found that the discharge intensity of the glow corona discharge is increased by 50 times. This is due to the negative glow area, which has a very high-intensity electric field.

The DDBOR current waveforms in positive and negative half-cycle are relatively smooth and only opposite in direction. This is because the DDBOR has the same discharge mode in the positive and negative half-cycle. Since the cathode and anode positions are reversed, the pulse direction is opposite.

To compare the effects of the silver layer, the current–voltage waveforms of a DBD reactor without the silver layer were measured, which are shown in Figure 5c. It can be seen that, in the negative half-cycle, the number of current pulses in the green dashed part is lower than that in Figure 5a. This result can also be seen in the orange dashed part of the voltage curve. Therefore, it can be inferred that the SDBOR reactor has a higher discharge intensity.

**Figure 6:**
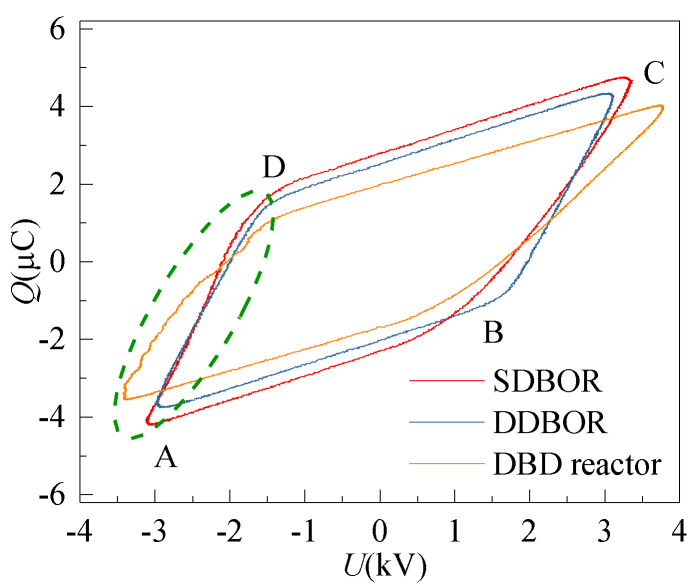
Lissajous Figure of SL-DBD (SDBOR, DDBOR) and DBD reactor without silver layer (applied voltage = 2.8 kV, discharge gap = 0.5 mm).

The discharge power of the reactor can be estimated by calculating the area of Lissajous figures, which are shown in Figure 6. It is clear that the area enclosed by the SDBOR curve is larger than that of the DDBOR, indicating that the discharge power of SDBOR is higher. The discharge power (*P*) is given by

(1) $$W=\int_{0}^{T}U(t)dq(t)$$

(2) $$P=\frac{S}{T}=f\times S$$

where *W* is the work in one cycle, *U* is the applied voltage, *q* is the electric charges, *T* is the time of a discharge cycle, *f* is the frequency of applied voltage, and *S* is the area of the Lissajous figure. With Equations (1) and (2), it can be found that when the applied voltage is 2.8 kV, the discharge powers for the SDBOR and the DDBOR are 190.5 W and 173.5 W, respectively.

In order to understand the role of the silver layer, the Lissajous figure of a DBD reactor without the silver layer (the structure is the same as SDBOR, but the dielectric layer is not covered by the silver layer) was recorded, which is the orange curve shown in Figure 6. Following the same calculation methods as mentioned above, it was found that when the applied voltage is 2.8 kV, the discharge power for the DBD reactor (without the silver layer) is 150.4 W, which is smaller than that of the SDBOR and DDBOR reactors. In general, the higher the slope of the B-C and D-A sections of the Lissajous figure is, the higher is the equivalent capacitance. It can be seen from the figure that the equivalent capacitance of the SL-DBD reactor is higher than that of the DBD reactor. In reactors, the higher the equivalent capacitance is, the higher is the discharge intensity. Therefore, it is clear that the silver layer can effectively improve the discharge intensity of the reactor. 

In addition, in the green dashed section of Figure 6, it can be seen that the orange curve (DBD reactor) is obviously deformed in the negative half-cycle, indicating that the discharge of the DBD reactor is unstable. On the contrary, the red and blue curves are very smooth, indicating that the discharge is generated stably in the SL-DBD reactor.

### 3.3. Effect of Discharge Modes on Electric Field Strength

**Figure 7:**
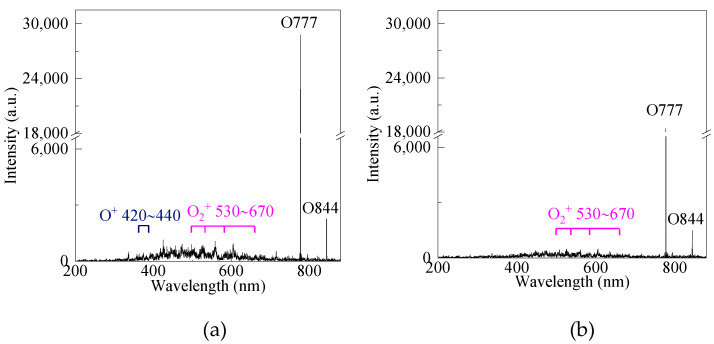
Emission spectrum of (**a**) SDBOR and (**b**) DDBOR (applied voltage = 2.8 kV mm, discharge gap = 0.5 mm).

The intensity of the electric field affects the concentration of ozone synthesis in the reactor. In this section, the effect of discharge modes on electric field strength is evaluated by the emission spectrum of the two reactors, as shown in Figure 7. It can be found that the spectrum intensity of SDBOR is higher than that of DDBOR. For example, the intensity of O^+^ and O_2_^+^ at 440 nm and 530 nm in SDBOR are 526 and 747, respectively. However, for DDBOR, it is less than 360. The intensity of O atom at 777 nm in SDBOR and DDBOR are 28,800 and 18,389, respectively. This means that the SDBOR produces more chemical products.

The reason for such results is that there are two discharge modes in SDBOR, which greatly increase the discharge strength. For DDBOR, however, there is only a streamer discharge mode. In SDBOR, during the formation of the intrinsic electric field, the electrons will be absorbed by the anode. Thus, the electric field is the sum of the applied electric field and the intrinsic electric field. In this experiment, the strong electric field can easily break down the dielectric layer. The use of the silver-coated dielectric layer can effectively avoid partial discharge and make the experiment stable. However, in DDBOR, the electrons are not absorbed by the anode because of the unexposed electrodes. Instead, charges are accumulated on the surface of the dielectric layer, which reduces the intrinsic electric field strength. Thus, the electric field strength in the SDBOR is higher than that in the DDBOR.

In addition, in the DBD reactor without the silver layer, there are no other peaks in the emission spectrum except for the O atom at 777 nm and 844 nm (Figure 7). The reason is that partial discharge occurs in the gap between the dielectric layer and the electrode, which reduces the electric field intensity in the normal working area. In addition, only a small amount of oxygen passes through the gap between the dielectric layer and the electrode. It is difficult to generate a high-intensity ionization excitation reaction in the gap.

### 3.4. Effect of Energy Density on Ozone Concentration

**Figure 8:**
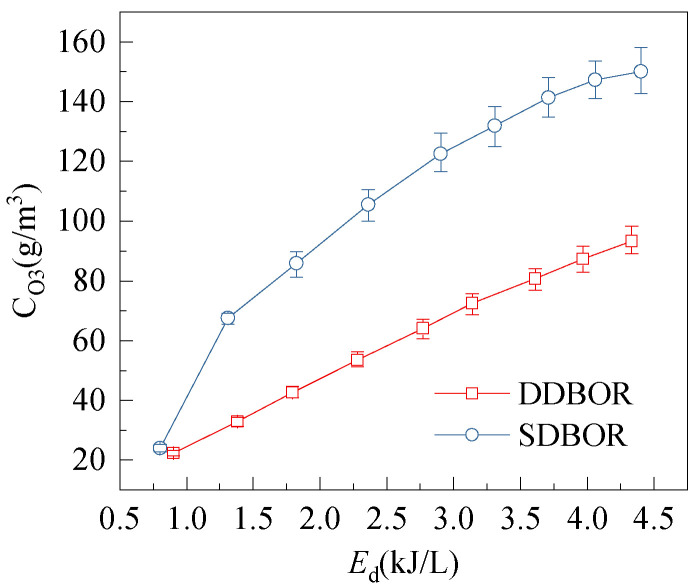
Dependence of ozone concentration on energy density (discharge gap = 0.5 mm, gas flow rate =3 L/min).

To compare the ozone synthesis per unit power for the two SL-DBD reactors, energy density is calculated. Theoretically, the energy density (*E*_d_) and ozone yield (*η*) are given by

(3) $$E_{d}=60\times P/F_{\mathrm{in}}$$

(4) $$\eta =60\times C_{O3}\times F_{\mathrm{in}}/P$$

where *C*_O3_ is ozone concentration, *P* is discharge power calculated by Lissajous figure, *F*_in_ is the flow rate of the feed gas, and the units of 60 is s/min. Figure 8 shows the dependence of ozone concentration on energy density. As can be seen from Figure 8, *C*_O3_ increases with the increase in *E*_d_ for both reactors. This is because, with the increase in *E*_d_, the discharge current increases; therefore, the concentration of ozone generated by the reactor increases. However, *C*_O3_ is higher for SDBOR under the same energy density. For example, when the *E*_d_ = 4.3 kJ/L, the ozone produced by SDBOR is 150 g/m^3^, and that in DDBOR is 93.3 g/m^3^. This is because the strength of discharge in SDBOR is higher, which indicates that the alternating streamer and glow corona produces more ozone at the same *E*_d_.

**Figure 9:**
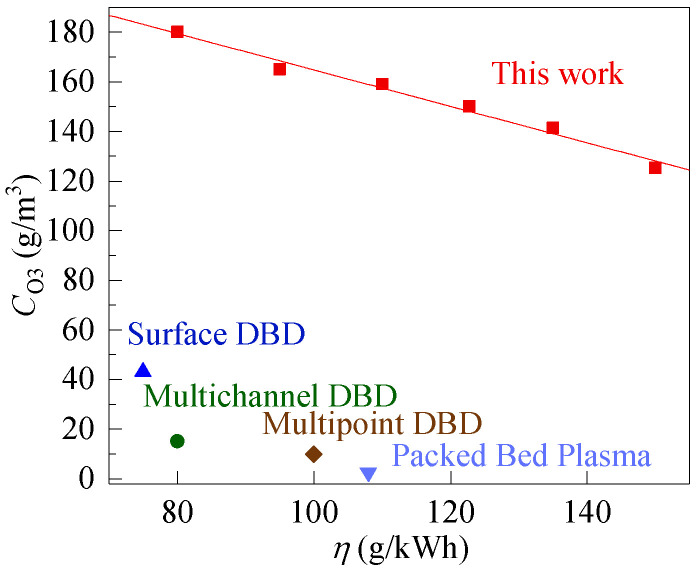
Comparison of *η* among various typical discharges for ozone synthesis.

Figure 9 compares the energy yield of ozone (*η*) and ozone concentration (*C*_O3_) in this work and those reported in the reference papers. It is clear that under the same energy yield of ozone, the ozone concentration in this work is the highest. For example, the ozone concentration is 10 g/m^3^ when the energy yield of ozone is 100 g/kWh in multipoint DBD [25]. For the reactor with a silver layer (this work), when the energy yield of ozone is 100 g/kWh, the ozone concentration is 162 g/m^3^, 16 times as much as that of the multipoint DBD. In multichannel DBD, when the energy yield of ozone is 80 g/kWh [26], the ozone concentration is 15 g/m^3^, only 8.3% of this work. For the surface DBD, when the energy yield of ozone is 75 g/kWh, the ozone concentration is 21.1% of this work [27]. In packed-bed plasma [28], when the energy yield of ozone is 108 g/kWh, the ozone concentration is 2.65 g/m^3^. The high-efficiency ozone generation of this work is contributed to the streamer and glow corona discharge generated alternately, whose discharge strength is 60 times as many as that of streamer discharge. For other reactors, however, there is only one discharge mode. It is clear that the reactor in this work significantly improves ozone synthesis efficiency.

### 3.5. Discussions on Discharge Mechanism

**Figure 10:**
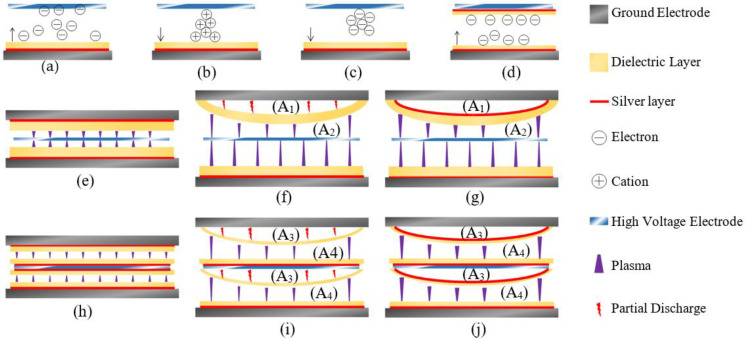
Schematic diagram of the mechanism of silver layer to improve discharge intensity. (**a**) schematic diagram of the electronic avalanche in SDBOR; (**b**) schematic diagram of the streamer in SDBOR; (**c**) schematic diagram of the glow corona discharge in SDBOR; (**d**) schematic diagram of the electronic avalanche in DDBOR; (**e**) internal structure of SDBOR; (**f**) schematic diagram of the discharge in single dielectric layer DBD reactor without silver layer; (**g**) schematic diagram of the discharge in SDBOR; (**h**) internal structure of DDBOR; (**i**) schematic diagram of the discharge in double dielectric layer DBD reactor without silver layer; (**j**) schematic diagram of the discharge in DDBOR.

Based on the above experimental results, the hybrid discharge processes (three stages) and the mechanism of silver-improved ozone synthesis under atmospheric pressure for the SL-DBD are put forward, as shown in Figure 10.

1. Stage 1
  When a new discharge begins in SDBOR, electrons start to move from the surface of the dielectric layer to the high-voltage electrode, as shown in Figure 10a [29]. The electrons collide with oxygen particles in the process of movement and produce a weak discharge. This process corresponds to section A-B in Figure 6. Normally, this process is called an electronic avalanche [30]. When electrons reach the high-voltage electrode, they are absorbed by the electrode [31]. However, in DDBOR, as shown in Figure 10d, the surface of the anode is covered with a dielectric layer so that electrons cannot be absorbed by the anode. This causes the number of displaced charges by SDBOR to be higher than that of DDBOR. The discharge parameters are the same, and the difference is the discharge mode. It can be seen from Figure 4 that the discharge intensity of SDBOR is higher than that of DDBOR.
2. Stage 2
  The second stage is the formation of streamer discharge. After the first stage, a large amount of cation is formed in the discharge gap. When the excitation electric field increases, the intensity of the electric field increases. The electric field formed by the charge and the excitation electric field are superimposed. The strong electric field ionizes the ambient gas and emits a large number of photons, causing photoionization, and forming a streamer discharge [32], as shown in Figure 10b. The arrow points to the direction of streamer discharge. This process corresponds to Figure 4a, the lower magnitude current plus in Figure 5a, and section B-C in Figure 6.
3. Stage 3
  When the negative high voltage is applied, the electric field near the high voltage electrode is distorted, which is caused by the superposition of the electric field. Due to the small mass, electrons quickly leave the strong electric field area and adhere to oxygen particles to form negative ions. After the electrons leave, the positive ions slowly approach the high-voltage electrode to form high-density positive ion clusters. The electric field between the positive ions and the high-voltage electrode is enhanced, forming the cathode potential drop region. A bright negative glow is formed behind the cathode potential drop region, forming a corona glow discharge [33], as shown in Figure 10c. During this process, the electric field intensity in the negative glow region is the highest, which corresponds to the P_1_ region in Figure 4b. This process corresponds to Figure 4b, the higher magnitude current plus in Figure 5a, and section D-A in Figure 6.

At the same time, the silver layer plays an important role in stably achieving hybrid discharge. Figure 10e is a schematic diagram of SDBOR. In SDBOR, the thickness of the dielectric layer is only 1.0 mm, and its stiffness is not enough to keep it flat. Concurrently, there will be a small gap (A_1_) between the electrode and the dielectric layer. When excitation voltage is applied to the electrode, as shown in Figure 10f, there is partial discharge in A_1_. The sum of the electric fields in A_1_ and A_2_ is the total applied electric field. However, A_1_ is not a normal working area and cannot produce ozone efficiently. In other words, it is equivalent to reducing the electric field intensity of the working area A_2_. After the silver layer is added, as shown by the red curve in Figure 10g, the potential difference in the A_1_ area can be zero to avoid partial discharge. On the other hand, the curved dielectric layer is equivalent to further reducing the discharge gap (less than 0.5 mm), resulting in a higher electric field intensity in A_2_.

Similarly, after adding a silver layer to the DDBOR, partial discharge in A_3_ can be avoided, and the electric field intensity in A_4_ is further increased, as shown in Figure 10h–j.

Through the above analysis, it is obvious that streamer discharge occurs in the positive half-cycle, and glow corona discharge occurs in the negative half-cycle. The silver layer can increase the electric field intensity of the discharge gap, and make the hybrid discharge more stable. Therefore, SL-DBD can produce ozone with higher efficiency.

### 3.6. Reliability Evaluation

**Figure 11:**
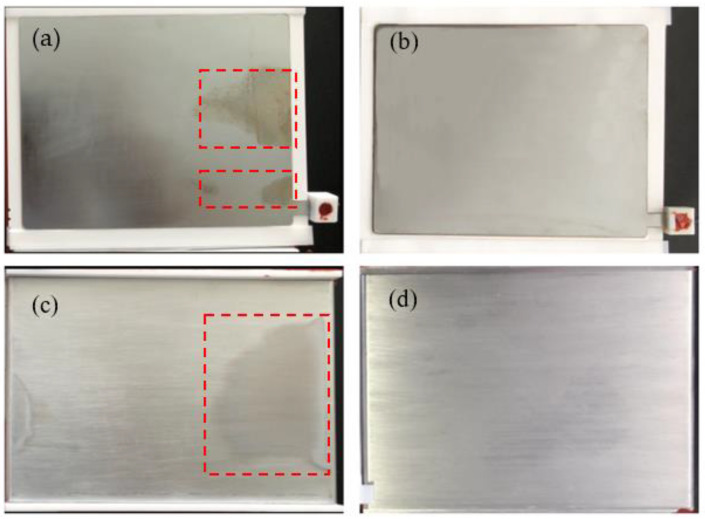
Morphologies of the high voltage electrode without silver layer (**a**) and with silver layer (**b**); morphologies of the ground electrode without silver layer (**c**) and with silver layer (**d**).

To evaluate the reliability of SL-DBD reactors, the morphologies of the dielectric-layer-covered electrodes with and without the silver layer were visually examined. As can be seen in Figure 11a,c, for the dielectric layer without the silver layer, there are a lot of metal oxide residues on the surface of the high voltage electrode and the ground electrode, which is most possibly due to partial discharge. Partial discharge is caused by a difference between the potential of the electrode and the dielectric layer, which breaks through the air gap and affects the normal discharge gap. Figure 11b,d shows the morphologies of the surface of the high-voltage electrode and ground electrode with the silver layer, respectively. It is clear that there is no obvious metal oxide residue, meaning that the silver layer on the dielectric effectively reduces the partial discharge phenomenon and promotes the generation of glow corona discharge.

**Figure 12:**
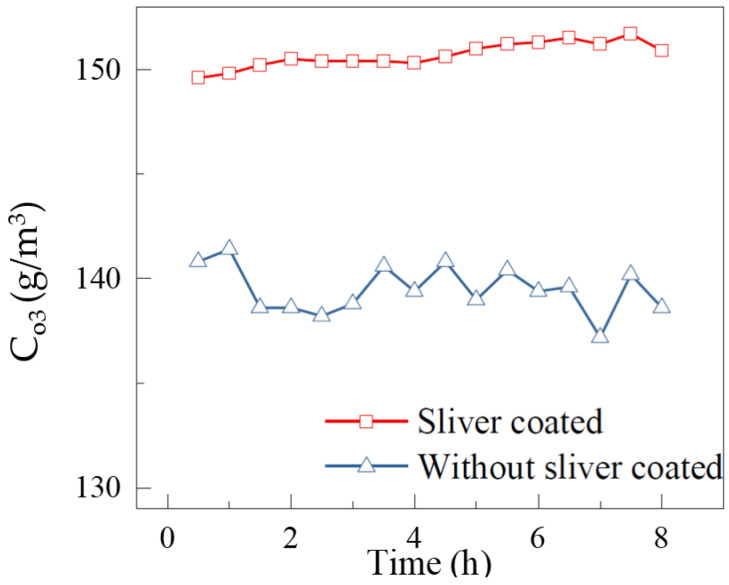
Dependence of ozone concentration on the running time (*F*_in_ = 1 L/min, *P* = 90 W).

To further examine the performance of the silver layer between the electrode and the dielectric layer, the two reactors work continuously for 8 h. The dependence of ozone concentration on the running time is shown in Figure 12. It can be seen that the *C*_O3_ varies from 149.6 g/m^3^ to 151.7 g/m^3^, for the silver layer reactor at *V* = 2.8 kV. As regards the reactor without the silver layer, the *C*_O3_ is 137.2 g/m^3^ to 141.4 g/m^3^. The *C*_O3_ of the reactor with the silver layer is stabilized at 150.6 g/m^3^ within 30 min, while the *C*_O3_ of the reactor without the silver layer is about 139.4 g/m^3^. It is clear that the efficiency of the reactor with the silver layer is higher than that of the reactor without the silver layer. Furthermore, the concentration of ozone produced by the reactor covered with the silver layer is much more stable. This suggests that the reactor with the silver layer works more reliably for a long time.

## 4. Conclusions

A novel SL-DBD ozone reactor was developed in this paper. By placing a silver layer on the surface of a dielectric layer, streamer and glow corona discharge were stably generated alternatively. The results show that adding a silver layer between the electrode and the dielectric layers prevents discharge from the contact surface of the electrode and the dielectric layer during 0.5 mm discharge. At the same time, the electric field intensity in the discharge gap is increased. SL-DBD reactor can effectively generate ozone with a concentration of as high as 150 g/m^3^. The spectral peak of O at 777 nm in SL-DBD is increased to 0.96, compared with 0.61 in a reactor with a streamer only. Compared with other reactors, the concentration of ozone generated by the novel reactor is increased by 10 times. The idea of placing a layer of silver between the electrode and the dielectric layer is an effective method for improving the electric strength for ozone synthesis and shows good stability in an 8 h durability test.

## Supplementary Materials

The following are available online at https://www.mdpi.com/article/10.3390/mi12111287/s1, Figure S1. Main circuit of IGBT inverter. Figure S2. PWM modulation circuit. Figure S3. Drive circuit of IGBT. Figure S4: Corresponding ICCD images of SDBOR.
