# Supplementary material for: A Novel Dielectric Barrier Discharge (DBD) Reactor with Streamer and Glow Corona Discharge for Improved Ozone Generation at Atmospheric Pressure
Source: Micromachines (Basel). 2021 Oct 21;12(11):1287. doi: 10.3390/mi12111287 (PMC8623020; doi:10.3390/mi12111287)
Supplement: Supplementary file 1 [file micromachines-12-01287-s001.zip › micromachines-1396076-supplementary.pdf]

A Novel Dielectric Barrier Discharge (DBD) Reactor with Streamer and Glow Corona Discharge for Improved Ozone Generation at Atmospheric pressure

Pu Liu, Yongxin Song and Zhitao Zhang

Design of IGBT inverter

The main circuit of the insulated gate bipolar transformer (IGBT) inverter is shown in Figure S1, including rectifier circuit, soft start circuit, filter circuit, and IGBT inverter circuit.

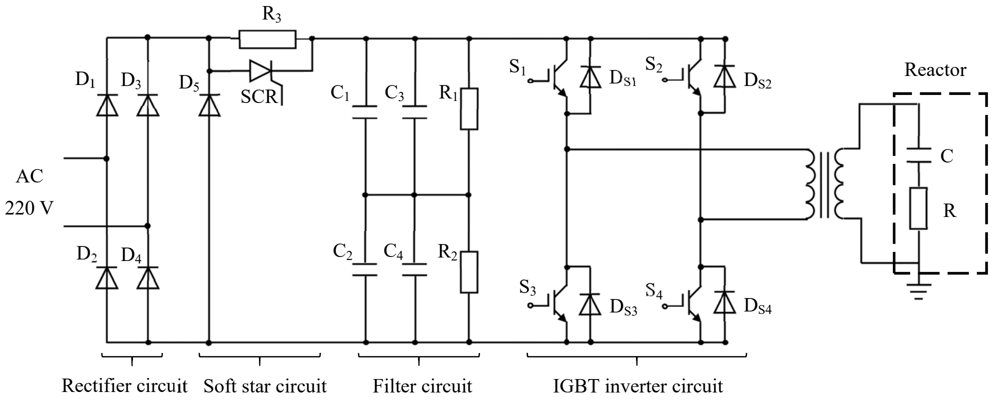

Figure S1. Main circuit of IGBT inverter.

The rectifier circuit uses the SKD62/18 rectifier module, which can adapt to AC and three-phase power. The rated voltage is 1800 V, and the rated current is 60 A.

The power supply soft start circuit is a high-power thyristor (SKKH106/16E). The rated voltage is 1700 V, and the rated current is 108 A.

The filter circuit uses two electrolytic capacitors (C_1, C_2) with a capacity of 2200 μF and a voltage of 450 V in series as the filter capacitors. The function of the filter capacitor is to reduce the pulse of the DC voltage and ensure a more stable output of the rectifier circuit. Each electrolytic capacitor is connected in parallel with a ceramic capacitor (C_3, C_4), with a capacity of 0.01 μF and a voltage of 1000 V. At the same time, a resistor (33 kΩ, 2 W) is connected in parallel. Its function is to eliminate the energy stored in the filter capacitor after the power supply is cut off.

The IGBT inverter circuit consists of 4 IGBT modules (SKM200GB176D) to form a full-bridge conversion circuit. The IGBT module of the opposite bridge arm is excited by the driving circuit in pulse with modulation (PWM) modulation, which converts the DC input into a square wave AC voltage of the required frequency.

The PWM modulation circuit uses SG3524 as the core component, and the circuit is shown in Figure S2. The pulse width is adjusted by the external circuit resistances R_1 and RP_1, and the output frequency is adjusted by the external circuit resistances R_2 and RP_2.

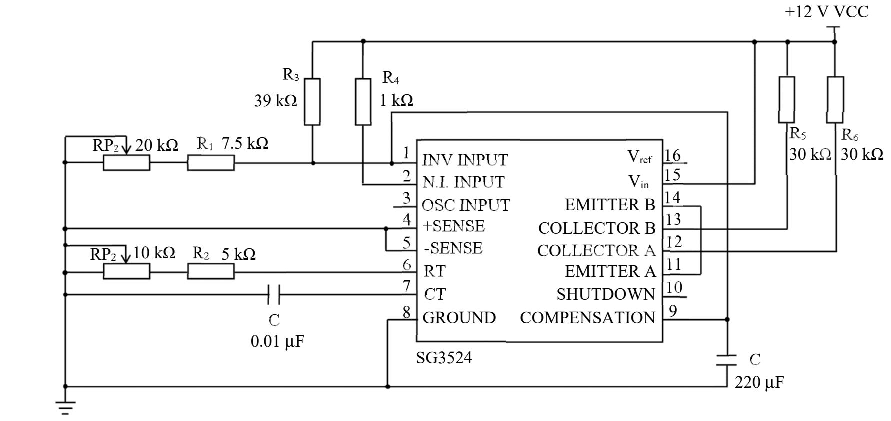

Figure S2. PWM modulation circuit.

The IGBT drive circuit uses the IGBT dedicated drive module VLA517-01R as the core device, and the drive circuit is shown in Figure S3.

A 6.2 V Zener diode D_4 with a power of 1 W is connected in parallel outside the VLA517-01R, and the power supply voltage is increased to 21 V. So the IGBT can be driven and turned off more reliably.

In addition, the fast recovery rectifier diode D_1 is connected in series between the overcurrent detection terminal of the drive circuit and the drain of the IGBT, which has a very important influence on the overcurrent protection of the IGBT. The forward conduction voltage drop is 3 V to increase the saturation conduction voltage drop.

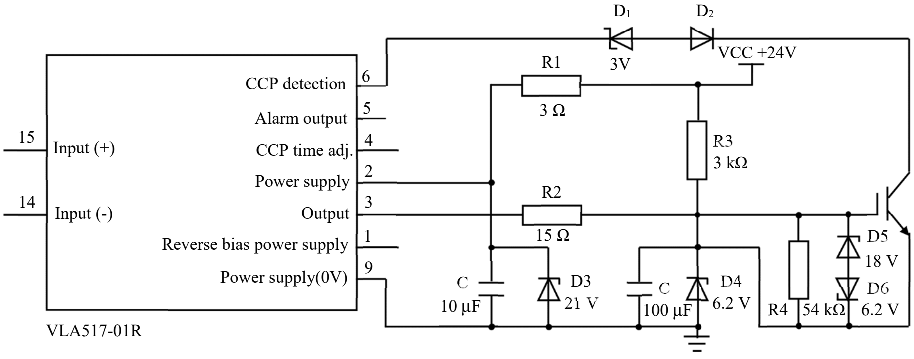

Figure S3. Drive circuit of IGBT.

The corresponding ICCD images of SDBOR

The synchronized trigger was used to take the single discharge image in SDBOR, as shown in Figure S4.

The current–voltage waveform of SDBOR is shown in Figure S4a. In SDBOR, the discharge waveform is different in the positive and negative half-cycles.

As shown in Figure S4b, the discharge mode is streamer discharge during the falling phase of the positive half-cycle of the current.

In the rising phase of the negative half-cycle of the current, the discharge image is shown in Figure S4c, and the discharge mode is corona discharge.

In the falling phase of the negative half-cycle of the current, the discharge image is shown in Figure S4d. The discharge mode is glow discharge, consisting of P_1 (negative glow area), P_2 (Faraday dark area), and P_3 (positive column area).

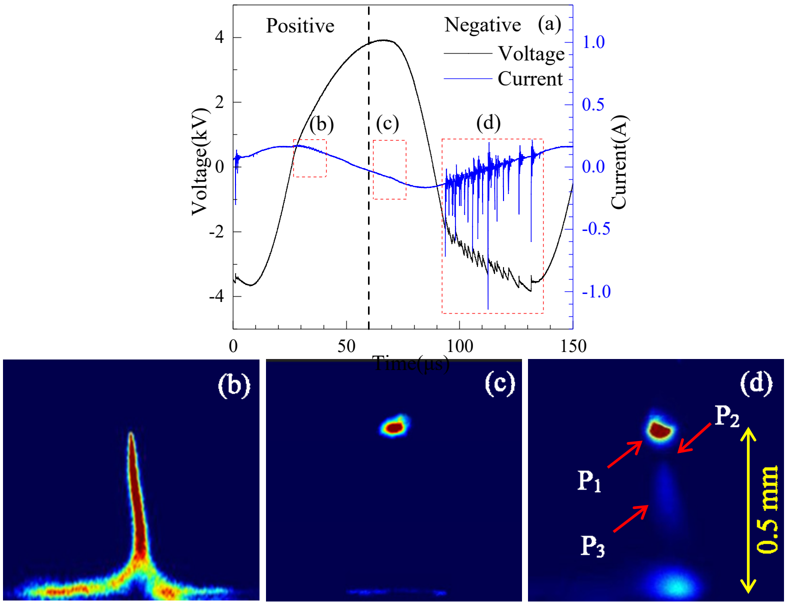

Figure S4. Corresponding ICCD images of SDBOR.
